# Outpatient management of REM sleep behavior disorder case in Brunner syndrome

**DOI:** 10.1192/j.eurpsy.2023.2341

**Published:** 2023-07-19

**Authors:** E. Cesari, I. Ochandiano, J. I. Mena, S. Salmeron, C. Gaig

**Affiliations:** 1 Department of Psychiatry and Psychology; 2 Department of Neurology and Sleep Center, Hospital Clinic de Barcelona, Barcelona, Spain

## Abstract

**Introduction:**

Brunner syndrome is a recessive X-linked disorder characterized by impulsive aggressiveness and mild mental retardation associated with Monoamine Oxidase – A (MAOA) deficiency (Brunner et al. Science 1993; 262 578-580).

**Objectives:**

To present a REM sleep behavior disorder (RBD) case in a patient with Brunner syndrome.

**Methods:**

The present study is a case report of a patient followed in our hospital’s outpatient care. We also searched for previous case reports of sleep disorders and other clinical features in Brunner syndrome using a pubmed query.

**Results:**

A 46-year-old Spanish male, diagnosed with Brunner syndrome due to the mutation c.1438A>G/iVS14-2 A>G, a loss-of-function mutation in the X-linked MAOA gene. He suffers from mild mental retardation and psychotic disturbances treated with SSRI and antipsychotic drugs. The patient was referred to our outpatient care to assess his sleep abnormal behaviors. He had been presenting with episodes of sleep-related vocalization and complex motor behaviors during sleep for the last 3 years, correlating with dream mentation. His relatives recounted episodes of talking, screaming, gesturing, kicking, falling out of bed and crying during sleep. Dream content referred by the patient was often related to persecutions, attacks and fights.

Polysomnography revealed vocalization and gesticulation during REM sleep compatible with the diagnosis of RBD. The addition of clonazepam to his treatment at doses of 1-3 mg per day achieved significant clinical response of the sleep disorder.

**Conclusions:**

The clinical presentation suggested the diagnosis of RBD case in a patient with Brunner syndrome. Although sleep disorders are not one of the most important or frequent clinical features in Brunner syndrome, they are described in the literature and can significantly affect the patient’s quality of life. To our knowledge, this is the first report about clinical management of RBD case in Brunner syndrome.

**Disclosure of Interest:**

None Declared

